# Frailty and the Risk of Polypharmacy in the Older Person: Enabling and Preventative Approaches

**DOI:** 10.1155/2020/6759521

**Published:** 2020-06-29

**Authors:** Martin C. Nwadiugwu

**Affiliations:** ^**1**^ Faculty of Health and Sports, University of Stirling, Stirling FK9 4LA, UK; ^**2**^ Department of Biomedical Informatics, University of Nebraska, Omaha 68182, NE, USA

## Abstract

Frail older people have an inherent risk of polypharmacy due to the need to treat multiple comorbidities, thus leading to various negative effects on their health due to the adverse actions from the drugs. This issue was discussed from a person-centered perspective, highlighting the category of frail older adults who are at a higher risk. Appropriate medication reconciliation in this population with useful prescribing tools (Beers and START/STOPP criteria) to minimize polypharmacy and to provide alternative prescriptive intervention could go alongside primary care to reduce the extent of frailty and polypharmacy. Reducing delayed referrals and extended hospitalization with electronic health record systems and using the signs of frailty from the Electronic Frailty Index (EFI) to predict polypharmacy for frail older persons are preventative approaches that proactively respond to frailty associated with the risk of polypharmacy.

## 1. Background

Frailty is a progressive condition characterized by a decline in cognitive, emotional, and physical ability resulting from the accumulation of gradual health deterioration [1] that leads to hospital admissions and multiple prescriptions which have not resulted in the best outcome [2]. Medication usage in older people increases with age due to associated comorbid conditions and the need to better manage overall outcomes [3]. Poor correspondence and communication between several health care providers could lead to the error of multiple prescriptions leading to polypharmacy, greater risk for adverse drug events, cognitive and functional impairment, falls, and adherence problems which can adversely impact the health of older persons and lead to higher risk for frailty [4, 5].

Frailty is associated with multiple health deficiencies [2], which suggests the need for treatment with multiple medications specific to the disease. Fortin et al. [6] suggested that multimorbidity is present in about 98% of the older population. This concurs with the cumulative deficit model of frailty that states that as people age, they acquire health deficits which confer increasing health risk [7]. As many diseases require treatment with medications, the increasing risk of side effects from medication is associated with frailty [8]; hence, older people who are frail are likely to suffer from the hazards of polypharmacy.

Polypharmacy is a defined risk for frailty [9]. It is “the use of multiple medications and/or the administration of more medications than are clinically indicated, representing unnecessary drug use” [9]. It is associated with the use of inappropriate medications that are either contraindicated or pose a high risk to older persons [9, 10]. The severity of frailty leads to the increasing intake of multiple medications which although may be recommended in the disease-specific guidelines, could be inappropriate for the older person when person-centered care is taken into account [11].

There is currently no consensus as to the multiple medication use that amounts to polypharmacy because of the need to treat presenting multiple comorbidities concurrently with combinations of drugs [12]. In estimating the combination of drugs that amounts to polypharmacy, some have said it is taking between 2 and 9 medications and others say it is the use of 5 or more medications and that hyperpolypharmacy amounts to the use of ten or more medications concurrently on a daily basis [12, 13]. Another definition deemed more appropriate is the use of medications not clinically indicated [10]. This definition details that some medications are clinically not needed and defines the term as the usage of redundant and unnecessary medication.

A person-centered approach to frailty focuses on knowing the frail older person, their values, and relationships, and seeing beyond their immediate needs has been suggested to be a proactive response to frailty [11, 14]. This is especially important in relation to the risk of polypharmacy, as the addition of a new medication for older people with frailty may trigger sudden changes in their mental and physical health [11].

This essay studies the associated risk of frailty using the cumulative deficit model which identifies frailty as an accumulation of health deficiencies [7]. Frailty will be discussed in relation to the risk of using multiple medications among frail older people from a person-centered perspective. It highlights the category of frail older persons more at risk and explains how a Comprehensive Geriatric Assessment (CGA) and a reduction in patient waiting time to access health care reflect person-centered approaches. Three preventative approaches, namely, efficient electronic medical record system, useful prescription aids (Beers and STOPP/START criteria), and social prescribing, will be explored to understand frailty in the older person in relation to the risk of polypharmacy, and optimization of functional capacity.

## 2. Frailty in the Older Person in relation to Polypharmacy

Current definitions of frailty place emphasis on the condition as a vulnerability characterized by loss of physiological reserves, emotional disorders, and inability to withstand acute illness [15, 16]. The cumulative deficit model by Rockwood [7] defines frailty as a situation where deficits outweigh the assets (well-being and functional capacity), while another definition by Fried is based mainly on five phenotypic criteria of physical dysfunction which includes low energy, low grip strength, slow walking speed, unintentional weight loss, and low physical activity [17]. Rockwood [7] operationalized a frailty index of 70 items characterized by many conditions (disability, cognitive and physical impairments, geriatric conditions, and psychosocial risks) that could lead to increased risk of developing adverse drug reactions (ADRs) [18].The physical manifesting factors or deficits of frailty are more likely to be identified and treated by clinicians than psychosocial factors, which raises concerns on the overall awareness of the issue, and is further complicated by the fact that a formal consensus on the definition of frailty is lacking [19].

Frailty is complex in the manner it occurs, and there are no general treatments. Medicinal interventions need to be person centered to examine the situation from the perspective of the person with the condition [19]. Person-centered care emphasizes the need for services to better engage with and be responsive to individual needs [20]. Although in the literature similar terms such as “patient-led” and “client-directed” have been used to refer to person-centered care with different emphasis [20], a common definition is that it prioritizes understanding the person's unique interpretation and experience of the illness [21] and taking cognizance of the decision made by the frail person in a positive care relationship [22, 23].

Frailty has been described as one of the factors which make polypharmacy more likely, as it is difficult for frail older persons with comorbid conditions to prevent the need for new prescriptions in ongoing clinical management [16]. The prevalence of polypharmacy is fueled by salient and nonspecific symptoms at hospital admissions [24]; moreover, the strong correlation between frailty and polypharmacy among older individuals and the risk for polypharmacy is high [25]. A descriptive cohort study to assess frailty, total number of health problems requiring treatment, geriatric problems, and medication among 250 patients aged above 65 years revealed that polypharmacy among frail patients had more significant health problems, longer hospital stays, and five times greater risk of readmission than patients without frailty and polypharmacy [24]. Another longitudinal and 8-year follow-up study by Veronese et al. [25] to investigate whether polypharmacy is linked with a higher incidence of frailty in a large cohort of 4402 North Americans at high risk of, or having, knee osteoarthritis, found that the incidence of frailty was significant in those taking 4–6 medications and 6 times higher in people taking 7 or more medications. These evidence-based studies suggest that taking more medications than are clinically indicated does not translate to better health for older people who are frail.

In addition, a study [13] involving 1705 community-dwelling older men that sought to identify those at risk of different adverse outcomes found that the use of 6.5 medications daily was associated with the risk of frailty and that the risk of being frail increases by 27% when the number of medication increases by one. This increase in medication leads to a 15% rise in the risk for falls and early mortality [13]. Thus, this suggests that polypharmacy and the risk for frailty are not only correlated but associated with other geriatric conditions which overtime could predict severe outcomes. The highlighted evidence establishes the relationship between the two and shows a rise in the risk for being frail in polypharmacy and hyperpolypharmacy compared with no polypharmacy.

It is important to prevent adverse events in order to create an environment where frail patients could thrive [26]. Budnitz et al. [27] reported that adverse drug reactions involved in prescription medication were common in one-third of emergency visits by older people. In 1996, Goldberg et al. [28] also reported that patients who took two drugs at the same time were found to have a 13% risk of adverse drug events, while those who took four and seven or more medications concurrently were 38% and 82% respectively at risk. The reports reveal that the more drugs one consumes, the higher the risk of adverse events due to a change in pharmacokinetics, drug-drug interaction, and drug interaction with living systems [29] and is a concern for frail older people who combine multiple medication concurrently [25, 30]. Therefore, it could be counterproductive to administer a large number of medications for multimorbid geriatric conditions without a comprehensive assessment. The comprehensive geriatric assessment (CGA) is a person-centered approach that could be used to reduce the extent of frailty associated with the risk of polypharmacy because it concentrates on medication that produces benefit but avoids those most likely to cause harm [19]. It is a holistic medical review involving multidimensional assessment and dialogue with older frail persons to understand their difficulties with activities of daily living and to create a comprehensive road map for care using evidence-based aids such as Beers criteria and STOPP/START criteria to improve prescribing [11, 31]. The comprehensive assessment optimize care by providing an alternative to medication when it is not vital and giving the patients the chance to prioritize care most appropriate for them.

The extent of frailty is associated with increasing number of health deficits that leads to frequent hospitalization and nursing home admission of older patients, increasing the risk for polypharmacy [32]. Nursing home residents are said to have the highest rate of polypharmacy because of the potential for increased diagnoses that correlates with increased health deficiency [33]. About 60% of home residential older persons (>75 year) in Denmark use more than 3 medications [34]. In Italy, 52% of hospitalized older patients take an average of 4.9 medications; in the United States, about 50% of hospitalized older patients take at least 7, while in Austria, 58% consume an average of 7.5 medications [32, 35, 36]. The distribution shows a high percentage of multiple drug use among hospitalized older persons compared with independent living residents.

Frail olderpeople feel insecure when they do not have the opportunity to contact a physician to communicate suspected worsening conditions [37]. It is important to note that effective patient communication [26] and a reduction in patient waiting time to access health care are person-centered approaches that enable frail older persons to have access to health care [38]. Short periods of consultation with a physician due to shortage of practitioners would likely not allow for a holistic medication review and comprehensive geriatric assessment (CGA) [39]. More health providers are needed to reduce patient waiting time and to routinely review older adult medication to prevent avoidable polypharmacy [40].

Consequently, it could be suggested that polypharmacy increases with advancing age [10] due to accumulation of deficits. Global estimation has it that 44.2–57.7% of adults aged 65 years or more are on at least 5 different drugs and 9.1–23.2% on 10 or more different drugs [30, 41]. There is no consensus on the increasing risk of polypharmacy among adults in their fourth age (≥85 years) while Jyrkkan et al. [42] and Haider et al. [43, 44] found that being in the oldest old category is a high risk factor, Onder et al. [45], Kim et al. [46], and Anja et al. [41] associated it with a lower risk of polypharmacy. Targeting factors that reduces the extent of frailty will lead to a reduction in polypharmacy and the accumulated deficits [47].

## 3. Preventative Approaches

An important objective of person-centered care for frail older people is to proactively respond to frailty to reduce the occurrence of polypharmacy [11]. To achieve this, the choices of older adults will have to be respected with more consideration of them as partners in their own health care [14]. Recommended preventative approaches include an efficient electronic medical record system, useful aids for appropriate prescription writing, social prescribing, and optimizing functional capacity by intravenous delivery of human allogenic mesenchymal stem cells.

A detailed electronic medical record would facilitate regular comprehensive review of older patient's prescriptions from healthcare providers and allow the patients to be more engaged in their own care [26]. Many physicians have little information about the patient's previous medical history and may prescribe without prior knowledge of what other physicians had recommended [48, 49]. An accessible electronic medical database would assist physicians in prescribing new drugs, reviewing medication efficacy, and to discontinue certain medications when alternatives with lesser adverse events exist [29]. This person-centered management program could be a way that proactively responds to frailty [26], to prevent avoidable polypharmacy and high risk for adverse drug events arising from multiple physicians' prescription [48].

Additionally, the electronic medical record allows access to an Electronic Frailty Index (EFI) which is used to find out and foretell adverse events for frail older patients [50]. It consists of 36 signs of frailty and a range of values that can be used to detect no frailty and mild-to-severe frail conditions. For example, older people with 12 signs of frailty would be classified as having the intermediate form [50]. Signs of frailty from the Electronic Frailty Index could be used to predict polypharmacy and adverse drug effects. Another advantage of the electronic medical record system is that it could prevent older people from being subjected to extended hospitalization and delayed referrals due to a complex medical history, as a reduction in time spent in hospital care reduces further frailty complexity and drug intake [32]. The advantages of EHR can be maximized if physicians can easily use it within a short period of time. Habboush et al. [51] reported that physicians spend about 49% of their working hours on indirect patient-related tasks such as EHR documentation and chart review. Development of more user-friendly EHRs and proper medical education for physicians on its usage could translate into more physician time with patients [51].

Another strategy that could mitigate worsening frail conditions and halt adverse drug events is social prescribing. Social prescribing is a way of referring patients to community groups and services for practical and emotional support [52]. It expands the options available to healthcare providers and gives patients the opportunity to form new relationships and take part in their own self-management [53, 54]. Since it has been reported that 8 or more diagnoses increases the risk of polypharmacy [41], social prescribing could go alongside with primary care to reduce polypharmacy [55], by connecting frail older people in healthcare services to community support providing a range of unorthodox prescriptive interventions such as volunteering, participating actively with exercise, and performance groups to foster social connectedness [52, 55–58]. An international consensus report on frailty in 2013 supports the fact that frailty could be treated to reduce polypharmacy by promoting physical activity with regular exercises, to optimize the functioning of the oldest old [59].

In a randomized and double-blind study by Tompkins et al. [60], results suggested that intravenous delivery of human allogenic mesenchymal stem cells in frail older people for bone and tissue repair, better physical performance, and resistance to inflammation is a possible way of reducing accumulated health deficits to optimize the functioning of the oldest old. The study which involved 30 older adults—10 in each of the three groups (two treatment and one control)—revealed a decrease in inflammatory markers (e.g., TNF-*α*) in the treatment group [60] and is consistent with similar findings by Schulman et al. [61]. A drawback of this study is that more reliable evidence is needed because of the small sample size and the lack of clarity on its application. However, the findings provide evidence that the extent of frailty and polypharmacy could be reduced by a reduction in the deficits and intake of more drugs (e.g., anti-inflammatory drugs) [62].

Furthermore, inappropriate prescribing is a concern for older people who are frail because they have altered pharmacokinetics that decreases the liver and kidney function as a result of reduced hepatic and renal clearance and altered pharmacodynamics that increases sensitivity to several drugs [2, 63]. This is more worrying as about 50% of older persons take drugs that are not necessary [33, 64]. Improved medication prescription using aids such as Beers criteria and the STOPP/START criteria can prevent unnecessary medication usage from a person-centered perspective by taking the contraindications and comorbidities of the frail individual into account [11].

The American Geriatrics Society (AGS) 2015 Beers criteria is a prescription aid focusing on drug monitoring to help frail older persons and healthcare providers, especially physicians, to choose a better combination of prescription medications, assess health outcomes among older patients, and evaluate the proper use of the selected drugs [65]. The screening tool of older people's prescriptions (STOPP) and the screening tool to alert right treatment (START) is another widely used prescription aid [66] that aims to avoid omissions and inappropriateness in prescription and has been reported to reduce the risk of adverse drug effect by 9.3% within three days of hospital admission [64]. The STOPP criteria for frail older persons (>65 years) is largely associated with adverse drug events and recommend against taking medications to treat symptoms unassociated with selected illnesses; however, the START criteria allows medication to be taken only when there is a documented history of the indicated illness and when more suitable drugs are contraindicated [67].

In essence, the prescription aids are part of the comprehensive geriatric assessment that takes the cumulative health deficit of the frail person into account and prescribes medication by understanding their history and lifestyle from a person-centered perspective [19, 26]. Using these benchmarks, healthcare providers can routinely review prescription drugs for older patients with multiple diagnoses and ensure prescribed drugs are clinically safe and more effectively reduce the risk of adverse events [26].

## 4. Conclusion

Frailty results from the accumulation of multiple age-related health deficiencies that require drug therapies. Causality exists between frailty and polypharmacy, and the risk for polypharmacy among frail older persons is high because of the need to treat multiple age-related disease conditions. The intake of multiple combinations of drugs increases the chance of accumulating health deficits arising from the side effects of drugs, resulting in poor health outcomes and increased risk of developing adverse drug reactions (ADRs).

A reduction in accumulated health deficiencies using person-centered approaches that better engages with frail individuals has been suggested as a forward-looking response to frailty. Allowing frail patients to effectively communicate with healthcare providers by reducing the waiting time to access health care and providing a comprehensive road map for care through a comprehensive geriatric assessment are person-centered approaches that could reduce the extent of frailty and polypharmacy.

In addition, carefully reviewing medication regimens to provide alternative prescriptive interventions such as exercise and social participation could go alongside primary care in optimizing the functional capacity of frail older people using intravenous delivery of human allogenic mesenchymal stem cells. These preventative approaches could reduce the extent of frailty and polypharmacy. The use of electronic health records to provide a comprehensive evaluation of health status may lower the rate of delayed referrals and shorten hospital stays, which reduces further frailty complexity. Signs of frailty from the Electronic Frailty Index (EFI) can be used to predict polypharmacy for frail older persons, while useful prescribing tools (Beers and START/STOPP criteria) can serve to enhance the physician's medical judgment and are invaluable tools to minimize the risk of polypharmacy.
